# Resistance Mutations to Broadly Neutralizing Antibodies Destabilize Hemagglutinin and Attenuate H1N1 Influenza Virus

**DOI:** 10.3390/v18010032

**Published:** 2025-12-24

**Authors:** Guohua Yang, Po-Ling Chen, Samuel W. Rovito, Karine Minari, Haley N. Writt, Jennifer DeBeauchamp, Jeri Carol Crumpton, Lisa Kercher, Rebecca M. DuBois, Richard J. Webby, Charles J. Russell

**Affiliations:** 1 Department of Host-Microbe Interactions, St. Jude Children’s Research Hospital, Memphis, TN 38105, USA; guohua.yang@stjude.org (G.Y.); po-ling.chen@stjude.org (P.-L.C.); samuel.rovito@stjude.org (S.W.R.); haley.writt@stjude.org (H.N.W.); jennifer.debeauchamp@stjude.org (J.D.); jeri.crumpton@stjude.org (J.C.C.); lisa.kercher@stjude.org (L.K.); richard.webby@stjude.org (R.J.W.); 2 Department of Biomolecular Engineering, University of California–Santa Cruz, Santa Cruz, CA 95064, USA; kminari@ucsc.edu (K.M.); rmdubois@ucsc.edu (R.M.D.)

**Keywords:** influenza virus, hemagglutinin, monoclonal antibody, neutralization, antibody escape, viral fusion glycoproteins, protein stability, virus transmission

## Abstract

Because antigenic drift primarily generates amino-acid changes in the membrane-distal hemagglutinin (HA) head, broadly neutralizing antibodies (bNAbs) are being developed to target conserved epitopes in the membrane-proximal stem. Mutations to HA2 residue A44, a buried residue beneath the central stem epitope, in 2009 H1N1 viruses have been shown to cause resistance to stem-binding bNAbs. Here, we introduced A44V and A44T mutations into A/Tennessee/1-560/2009 (TN09) and A/Puerto Rico/15/2018 (PR18) and investigated their effects in cell culture, mice, and ferrets. In both virus strains, the mutations decreased HA and virus stability and decreased bNAb binding and neutralization in vitro. The mutations reduced pathogenicity and lung replication in DBA/2J mice. Ferrets were inoculated with PR18 wild-type (WT) or A44V virus, and the A44V mutation reduced day-1 and peak nasal virus titers. Airborne transmission in the A44V group occurred only after genotypic reversion (HA2-V44A) or acquisition of a distal re-stabilizing mutation (HA2-I77M). Compared to WT, an engineered PR18 virus containing both HA2 mutations (A44V and I77M) had similar growth and pathogenicity in mice in addition to decreased binding and neutralization by bNAbs. Overall, this work provides insight into the role of HA stability during HA stem-epitope remodeling that results in virus resistance to stem-binding bNAbs.

## 1. Introduction

Influenza viruses cause substantial morbidity and mortality in humans despite the availability of multiple vaccine platforms and antiviral therapeutics. The World Health Organization (WHO) estimates that influenza viruses cause approximately 1 billion infections, 3–5 million severe cases, and 290,000–650,000 deaths worldwide each year [1]. Endemic (“seasonal”) strains include two type A subtypes (H1N1 and H3N2) and one type B lineage [2]. Humans have little or no preexisting immunity to animal-origin influenza viruses, such as H5 and H7N9, which occasionally infect humans [3]. If these emerging viruses acquire human-adapted traits, they could cause a pandemic [4,5,6]. Because of antigenic drift and shift, broadly protective vaccines and therapeutics targeting multiple subtypes, or even drifted strains within a subtype, could greatly improve long-term influenza prevention and control [7,8].

**Figure 1:**
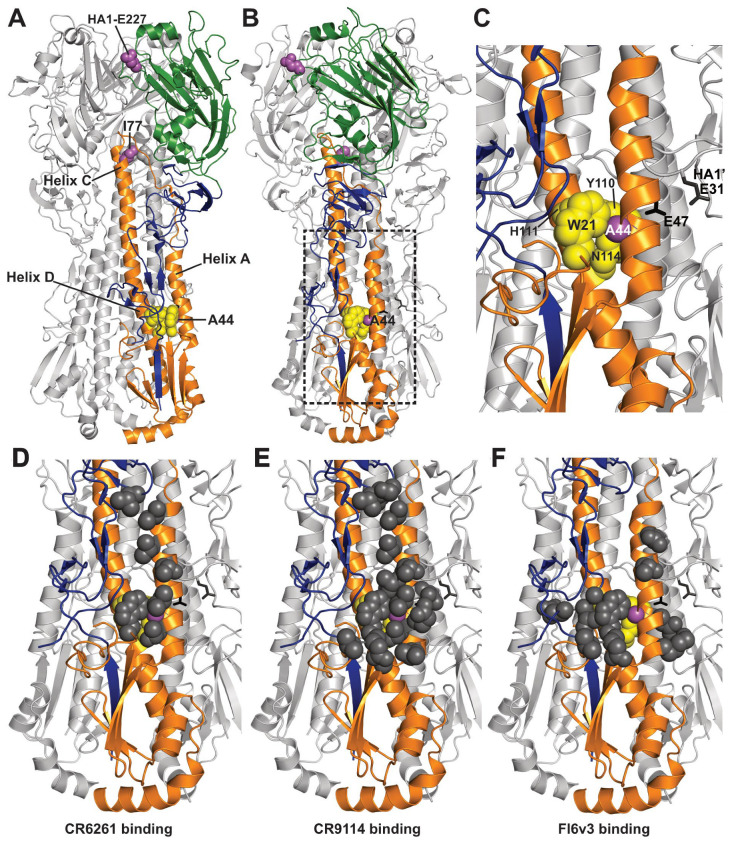
Structure of the H1N1 HA trimer ectodomain, including bNAb epitopes and resistance mutations. For clarity, two protomers are colored gray while one is color-coded as follows: HA1 stem (blue), HA1 head (green), and HA2 ectodomain (orange). (**A**) HA ectodomain with α-helices A, C, and D notated along with residues HA2-A44, HA2-I77M, and HA1-E227 shown as magenta spheres. The residues constituting a binding pocket for A44 (HA2 W21, Y110, and N114) are shown as yellow spheres. (**B**) 45-degree rotation of the HA trimer showing the packing of A44 (magenta) into its pocket (yellow spheres). The dotted rectangle defines the zoomed-in focus of panels (**C**–**F**). (**C**) Close-up view of A44 in addition to identification of residues HA2-E47 and HA1-E31. (**D**–**F**) Binding sites of bNAbs CR6261 (**D**), CR9114 (**E**), and FI6v3 (**F**). Structure 3UBE was used for modeling [20].

The hemagglutinin (HA) surface glycoprotein is the main antigen targeted by naturally acquired and vaccine-induced immunity [9,10]. After HA binds host–cell receptors and the virus is endocytosed, cleaved HA trimers cause membrane fusion [11]. Antibodies can block receptor binding and membrane fusion and can also mediate antibody-dependent cellular cytotoxicity (ADCC) [12]. Antigenic drift arises from sequence variation in immunodominant sites within the membrane-distal head domain of HA [13]. Consequently, next-generation vaccines and biologics aim to target more conserved regions, primarily the central stem epitope within the membrane-proximal “stem” or “stalk” domain [7,14,15]. For example, bNAbs CR6261, CR9114, and FI6V3 bind to the central stem epitope that contains residues in α-helices A and D [16,17,18,19] (Figure 1D–F).

The first influenza human challenge study using Good Manufacturing Practice (GMP)-produced virus under an Investigational New Drug application (IND) was conducted in 2012–2013 [21]. It aimed to determine the median infectious dose (ID_50_) of wild-type (WT) A/CA/04/2009 (H1N1) that produces mild to moderate disease in healthy adults. The inoculated virus contained one mutation in the 3′-untranslated region (UTR) and seven nonsynonymous mutations [22]. Three of these were in HA: K71N, A388V, and T451N (numbering from the post-translation methionine). Using Burke/Smith numbering [23], these correspond to K54N, A371V, and T434N. In H3 numbering [19], which will be used here, they correspond to HA1-K63N, HA2-A44V, and HA2-T107N.

The HA2-A44V mutation is central to the present study. HA2-A44 lies within the buried, non–solvent-accessible surface of α-helix A (Figure 1A). In the human-challenge study, the inoculated virus population contained approximately 45% WT (HA2-A44) and 55% HA2-A44V [22]. A follow-up study showed that HA2-A44V was selected in participants with high pre-challenge levels of stalk-binding antibodies, that the mutation conferred resistance to central-stem bNAbs (CR6261, CR9114, FI6V3, 70-1F02, C179, and CT149), and that the mutant virus had attenuated replication in mice but replicated similarly to WT virus in ferrets [24]. Thus, a buried point mutation at HA2 position 44 in α-helix A conferred resistance to stem-binding bNAbs while maintaining replication competence in ferrets, an established model for human influenza infection.

Several novel vaccines aim to induce broader protection than current influenza vaccines. One promising approach is sequential vaccination with chimeric HAs containing antigenically distinct head domains to enhance antibody responses to the conserved stem [25]. To measure potential antigenic drift in the stem and its effect on chimeric HA vaccination, six escape mutant A/Netherlands/602/2009 (H1N1) viruses were generated using stem-binding antibodies [26]. Four contained the HA2-A44T mutation (reported as A388T when numbered from the N-terminal methionine), which reduced both neutralization by stem-binding bNAbs and ADCC activity. Mice immunized sequentially with chimeric HA antigens having antigenically mismatched heads, to boost immune responses to the stem, were protected against challenge by a virus containing HA2-A44T. This shows the robustness of a vaccination strategy that induces polyclonal antibody responses to the stem region. Although the HA2-A44T mutation was attenuating in mice, it was not tested in ferrets [26].

The observation that HA2-A44 mutation confers resistance to stem-binding bNAbs represents a potential barrier to the long-term use of HA stem-directed therapeutics. Therefore, the present study investigated the impact of HA2-A44 mutations in a more recent H1N1 strain (A/Puerto Rico/15/2018, PR18) in addition to a 2009 H1N1 strain.

## 2. Materials and Methods

### 2.1. Cell Lines and Viruses

Madin-Darby Canine Kidney (MDCK, ATCC, CCL-34), human lung adenocarcinoma (A549, ATCC, CCL-185), African green monkey kidney (Vero, ATCC, CCL-81), and human embryonic kidney (HEK 293T, ATCC, CCL-11268) cells were obtained from the American Type Culture Collection (ATCC). MDCK and Vero cells were maintained in Dulbecco’s Modified Eagle’s Medium (DMEM) supplemented with 5% fetal bovine serum (FBS) and 1% penicillin-streptomycin (Pen-Strep). A549 cells were maintained in Ham’s F-12K (Kaighn’s Modification) medium containing 10% FBS. 293T cells were grown in Optimized Minimal Essential Media (Opti-MEM) with 10% FBS and 1% Pen-Strep. All cells were incubated at 37 °C with 5% CO_2_.

A/Tennessee/1-560/2009 (TN09) and PR18 viruses were rescued by reverse genetics. pHW2000 plasmids were cloned with each gene segment of TN09 and PR18 viruses. HA mutations in this study were introduced to pHW2000-TN09-HA and pHW2000-PR18-HA by using the QuikChange II XL Site Directed Mutagenesis Kit (Stratagene, Cedar Creek, TX, USA). During virus rescue, plasmids were transfected into a co-culture of MDCK and 293T cells, and then the transfection was further amplified in MDCK cells as described previously [27]. The rescued viruses were titrated in MDCK cells by 50% Tissue Culture Infectious Dose (TCID_50_) assay and/or plaque assay. All viruses were identified by whole genome sequencing.

### 2.2. HA and Virus Stability Assay

HA stability was determined by syncytia assay. Vero cells were infected with viruses at a multiplicity of infection (MOI) of 3 Plaque-Forming Unit (PFU)/cell. One hour later, virus-containing supernatants were aspirated from cells and replaced with fresh medium lacking L-(tosylamido-2-phenyl) ethyl chloromethyl ketone (TPCK)-treated trypsin (TPCK-treated trypsin). After overnight incubation, the supernatant was removed, and the cells were treated with 5 µg/mL of TPCK-treated trypsin for 15 min at 37 °C, followed by a 10-min treatment of pH-adjusted Phosphate-buffered saline (PBS) buffers at 37 °C. After pH treatment, the cells were allowed to recover in DMEM + 5% FBS medium for 3 h at 37 °C. The cells were fixed and stained by using Hema 3 kit (Fisher Scientific, Kalamazoo, MI, USA). Syncytia formation was quantified after using a benchtop light microscope to collect photomicrographs. The highest pH at which syncytia formation was triggered was defined as HA activation pH (or fusion pH) [28].

Virus stability was determined by acid inactivation assay. Virus aliquots were mixed with pH-adjusted PBS buffers (1:100) and incubated at 37 °C for 1 h. After treatment, the viruses were neutralized and then titrated in MDCK cells. The log_10_(virus titer) for each treated pH value was plotted in GraphPad Prism 10. Virus inactivation pH was calculated using the four-parameter logistic (4PL) curve [29].

### 2.3. Enzyme-Linked Immunosorbent Assay (ELISA)

Viruses (5–10 Hemagglutination units, HAU) were added to fetuin-coated 96-well plates (100 µL/well). Plates were sealed and incubated at 4 °C overnight. After virus coating, plates were washed 5 times with chilled washing buffer (1X PBST: 1X PBS with 0.1% Tween-20). 200 µL of blocking buffer (3% of Bovine Serum Albumin (BSA) in 1X PBST) was added to each well, and samples were incubated at 4 °C for 1 h. After blocking, the plate was washed with chilled washing buffer 3 times. bNAbs were serially diluted with blocking buffer, and then 50 µL/well of diluted bNAb was added to each well. After 3-h incubation at 4 °C, plates were washed with chilled washing buffer 5 times, and then 50 μL/well of diluted secondary antibody was added to each well. After 1-h incubation at 4 °C, plates were washed with chilled washing buffer 5 times, and then 100 µL/well of 3,3′,5,5′-Tetramethylbenzidine (TMB) substrate was added to each well. Plates were incubated in the dark at room temperature for 15–30 min, and then 50 µL/well of stop solution (3% H_2_SO_4_) was added to stop the reaction. The absorbance at 450 nm wavelength was read using a BioTek Synergy H1 (Agilent Technologies, Santa Clara, CA, USA). Four bNAbs targeting HA stalk region (CR6261, CR9114, FI6V3, and CT149; Creative Biolabs, NY, USA), and one antibody targeting the head region (2-12C) were used in this study.

### 2.4. Microneutralization Assay

MDCK cells were seeded in 96-well plates (3 × 10^6^ cells/plate) and incubated at 37 °C overnight. Tested antibodies were 2-fold serially diluted with infection medium using a starting concentration of 40 µg/mL. Diluted viruses (2000 TCID_50_/_mL_) were added to diluted antibodies (1:1) and then incubated at 37 °C for 1 h. 100 µL/well of virus-antibody mixture was added to washed MDCK cells and then incubated at 37 °C for 1 h. The mixture was removed, and the cells were covered by fresh infection medium (100 µL/well) and incubated at 37 °C for 3 days. The percentage of infection was normalized to the lowest antibody concentration, and the half maximal inhibitory concentration (IC_50_) was measured using a 4PL curve in GraphPad Prism 10.

### 2.5. Mouse Studies

For clinical symptoms of infection, groups of five eight-week-old female DBA/2J mice (Jackson Laboratories, Bar Harbor, ME, USA) were inoculated intranasally with TN09 (750 PFU) or PR18 (25,000 PFU). Weight change and survival were recorded daily for 15 days. Mice that lost 25% body weight were euthanized. To measure virus replication, 15 mice were intranasally inoculated with TN09 (750 PFU) or PR18 (25,000 PFU). To measure lung viral loads, groups of five mice were euthanized, and lungs were harvested at 2-, 4-, and 6-days post-infection (dpi). Lungs were homogenized, and viral loads were measured by TCID50 assay. Mice were randomly assigned to cages by blinded staff, and no animals were excluded from data analyses.

### 2.6. Ferret Transmission Study

Each virus was tested in 3 pairs of 5-month-old male ferrets (Triple F Farms, Gillett, PA, USA). Each pair had one donor ferret, one direct contact ferret, and one aerosol contact ferret for a total of 9 ferrets for each virus. Each transmission cage housed 3 ferrets and had two chambers separated by a pegboard wall. On day 0, a donor ferret was intranasally inoculated with 1 × 10^6^ PFU of viruses and placed in the left chamber of the transmission cage. On 1 dpi, a direct contact ferret was placed in the left chamber with the donor ferret, and an aerosol contact ferret was placed in the right chamber of the same transmission cage. The body weight and temperature of the ferrets were recorded every day until 16 dpi. The nasal wash of the donor ferrets was collected every second day from 1 dpi, and the nasal wash of direct contact and aerosol contact ferrets was collected every second day from 2 dpi. The viral load in nasal wash was measured in MDCK cells by using TCID_50_ assay. On 21 dpi, all ferrets were sacrificed, and the blood was collected for serological assay. The room temperature and humidity were also monitored during the experiments [29]. Ferrets were randomly assigned to cages by blinded staff, and no animals were excluded from data analyses.

### 2.7. Expression and Purification of HA Protein Ectodomains and Biolayer Interferometry [24]

Codon-optimized synthetic complementary DNAs (cDNAs) corresponding to the HA ectodomains of HA-WT, HA2-A44V, HA2-A44V/HA1-E227G, and HA2-A44V/I77M fused to a C-terminal Foldon trimerization domain, Avi-Tag, and 6x His-tag were cloned into a pcDNA3.1 derivative vector (GenScript) and maxiprepped. Chinese hamster ovary S (CHO-S) cells (Invitrogen, Waltham, MA, USA; product 514448) were grown at 37 °C and transfected with these plasmids via electroporation with the MaxCyte STX transfection system. Following transfection, CHO-S cells were resuspended in CD-OptiCHO media (Gibco, Grand Island, NY, USA; product 12681029) and were given a final concentration of 1 mM sodium butyrate and maintained at 32 °C, 8% CO_2_, 85% humidity, 135 rpm. CHO-S cells were fed CHO feed (CHO CD EfficientFeed A (Gibco, Grand Island, NY, USA; product A1023401) supplemented with 7 mM L-glutamine, 5.5% glucose, and 23.4 g/L yeastolate) every 24 h for 5–11 days. CHO-S cells were centrifuged, and the resulting supernatants were given 1X protease inhibitor cocktail (MilliporeSigma, Burlington, MA, USA; product 539137) and wash buffer (20 mM sodium phosphate, 0.5 M NaCl, pH 7.4) and were 0.22 µm filtered. Samples were purified by affinity chromatography using the HisTrap Excel column (Cytiva, Marlborough, MA, USA; product 29048586) equilibrated in wash buffer. After washing, the proteins were eluted with a linear gradient of wash buffer containing 500 mM imidazole. Coomassie-stained sodium dodecyl sulfate–polyacrylamide gel electrophoresis (SDS-PAGE) was used to analyze protein purity. Proteins were dialyzed into PBS pH 7.4 and flash-frozen in liquid nitrogen for long-term storage at −80 °C.

Biolayer interferometry (BLI) data were collected on an Octet RED384 using the Data Acquisition Software (version 11.1.1.19). Binding experiments were performed in freshly prepared and filtered kinetics buffer containing PBS (Sigma-Aldrich, St. Louis, MO, USA; product P4417), 0.5% BSA (Thermo Fisher Scientific, Waltham, MA, USA; product BP1600-100), and 0.05% Tween 20 (Thermo Fisher Scientific, Waltham, MA, USA; product BP337-100).

For the binding studies, monoclonal antibody (mAb) CR6261 (Diridavumab recombinant human monoclonal antibody, Invitrogen, Waltham, MA, USA; product MA5-42027) was diluted in kinetics buffer in series from 40 nM to 5 nM. mAb P1-05, produced as described previously [30], was diluted in kinetics buffer in series from 20 nM to 2.5 nM.

Octet^®^ HIS1K Biosensors (Sartorius AG, Göttingen, Germany; product 18-5120) were equilibrated for 1 h in kinetics buffer at room temperature. The Octet RED384 temperature was set to 24 °C and the plate shaking was 1000 rpm. Biosensors were immersed in kinetics buffer for a baseline of 60 s, loaded with 1 nm signal of HA protein (HA-WT, HA2-A44V, HA2-A44V/HA1-E227G, and HA2-A44V/I77M) in kinetics buffer for 300 s. Biosensors were then dipped into the kinetics buffer for 60 s to obtain a baseline reading. To measure association, biosensors were dipped into the wells containing mAb CR6261 or mAb P1-05 for 300 s. To measure dissociation, biosensors were returned to wells with kinetics buffer for 600 s.

The data were processed in the Octet Data Analysis HT software (version 11.1.1.19). Each curve was reference-subtracted, aligned to the average of the baseline, and aligned for inter-step correction through the baseline. The Savitzky–Golay filtering was applied to remove the high-frequency noise. At least 4 curves from each replicate were fit globally using a 1:2 bivalent analyte binding model. Average dissociation constants (K_D_ values) are reported as the mean of two independent experiments.

### 2.8. Statistical Analyses

All data were analyzed by using GraphPad Prism 10 software. *p* values < 0.05 were considered statistically significant. The binding affinity, IC_50_, growth curve in vitro, and viral load in mouse lungs were compared using ordinary two-way analysis of variance (ANOVA) followed by Tukey’s multiple comparison test. The virus replication in the nasal cavity was compared using an unpaired *t*-test with a Gaussian distribution.

## 3. Results

### 3.1. HA2-A44V and HA2-A44T Mutations Reduce bNAb Binding and Neutralization

**Figure 2:**
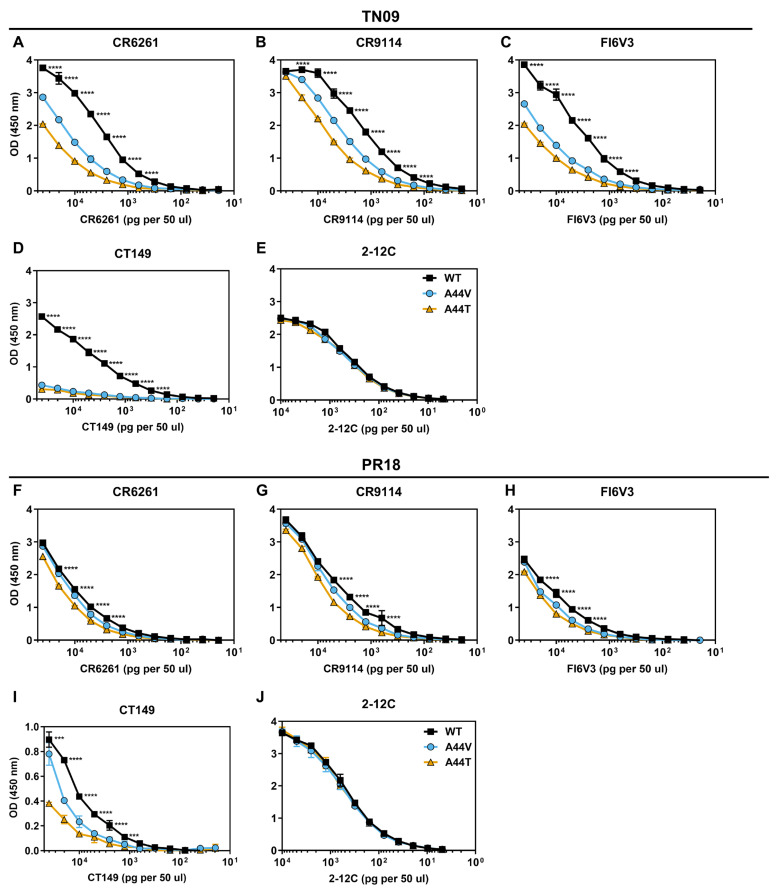
Binding of stem-binding bNAbs to TN09 and PR18 viruses. Binding of mAbs to virus was analyzed by ELISA assay. CR6261 (**A**,**F**), CR9114 (**B**,**G**), FI6V3 (**C**,**H**), and CT149 (**E**,**I**) are broadly reactive antibodies targeting the central HA stem epitope. 2-12C (**E**,**J**) binds to HA globular head and was used as a control. (**A**–**E**) Binding to TN09 viruses. (**F**–**J**) Binding to PR18 viruses. The data was from two biological replicates. The error bars are the average with standard deviation. Ordinary two-way ANOVA was used for statistical analysis, followed by Tukey’s multiple comparison test. *** *p* < 0.001, **** *p* < 0.0001.

Previous studies found that A/CA/04/2009 HA2-A44V and A/Netherlands/602/2009 HA2-A44T were resistant to stem-binding bNAbs [24,26]. Here, we generated six recombinant viruses. These included two genetic backgrounds (TN09 and PR18) and three HA variations (WT, A44V, and A44T). ELISAs were performed to measure binding of stem-binding bNAbs CR6261, CR9114, FI6V3, and CT149. Antibody 2-12C, which targets the HA head domain, served as a negative control. For TN09 viruses, CR6261, CR9114, and FI6V3 had the strongest binding to WT, followed by A44V, then A44T (Figure 2A–C). CT149 binding was equally low for TN09 A44V and A44T (Figure 2D). Thus, A44V and A44T mutations caused reduced binding to bNAbs in the TN09 background, consistent with findings from other 2009 isolates [24,26]. For PR18-strain viruses, differences in binding by CR6261, CR9114, and FI6V3 between mutant and WT viruses were statistically significant yet less pronounced than in the background of TN09 (Figure 2F–J).

Neutralization was measured for all six viruses. Comparing WT viruses, IC_50_ values were approximately 8-fold and 4-fold lower for PR18 than TN09 for CR6261 and CR9114, respectively (Table 1), indicating that these bNAbs had greater potency in the more recent strain. In both genetic backgrounds, A44 mutations increased IC_50_ values by approximately 2–3-fold relative to WT, showing resistance to neutralization by stem-binding bNAbs.

### 3.2. HA2-A44 Mutations Decrease HA and Virus Stability

**Figure 3:**
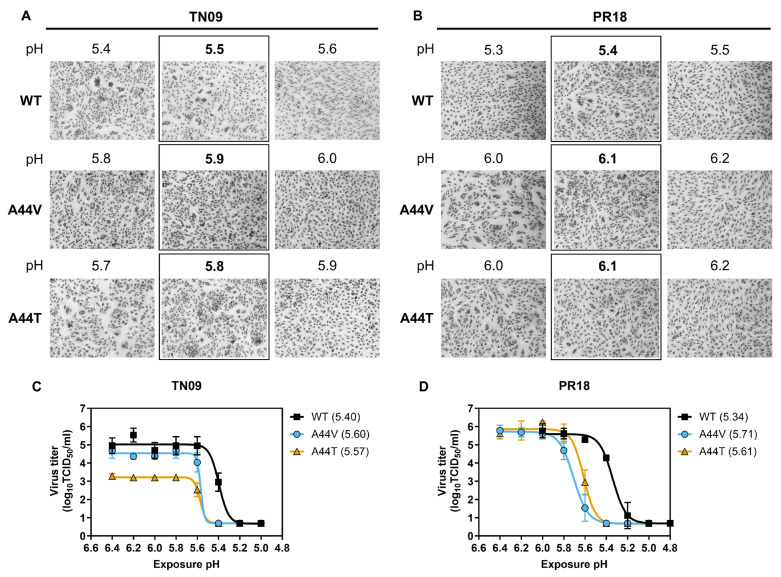
HA and virus stability. (**A**,**B**) HA stability as determined by syncytia in Vero cells infected by TN09 viruses (**A**) or PR18 viruses (**B**). Vero cells were infected with influenza virus and later treated pulsed with pH-adjusted buffers before re-neutralization and allowing pH-activated HA proteins to cause cell-to-cell fusion (syncytia formation). Activation pH was defined as the highest pH at which syncytia were observed (central panels). (**C**,**D**) Virus stability as determined by virus inactivation assay. Aliquots of TN09 viruses (**C**) and PR18 viruses (**D**) were treated with pH-adjusted buffers before re-neutralization and being subjected to TCID_50_ assay to measure the remaining infectivity as a function of pH. The virus inactivation pH values were calculated using a 4PL curve in GraphPad Prism 10. The error bars are the geomean with geometric standard deviation. Virus inactivation pH values are labeled in the legends.

The effects of the A44 mutations on HA stability were measured using a syncytia formation assay. In the TN09 background, the highest pH for syncytium formation was 5.5 for WT, while the A44V and A44T mutations increased this value to pH 5.9 and 5.8, respectively (Figure 3A). The PR18 viruses followed a similar trend, with WT having an activation pH of 5.4 and the mutant viruses having higher activation pH values of 6.1 (Figure 3B).

To measure viral stability, aliquots were incubated in buffers ranging from pH 5.0 to 6.4, re-neutralized, and assayed for residual infectivity by TCID_50_ (Table 2). For TN09, WT had a virus inactivation pH of 5.40, while A44V and A44T increased the virus inactivation pH to 5.60 and 5.57, respectively (Figure 3C). PR18 WT had a virus inactivation pH of 5.34, and A44V and A44T had increased values of 5.71 and 5.61, respectively (Figure 3D). Overall, the A44V and A44T mutations destabilized the HA protein, decreasing the acid stabilities of the viruses.

### 3.3. Virus Growth In Vitro

**Figure 4:**
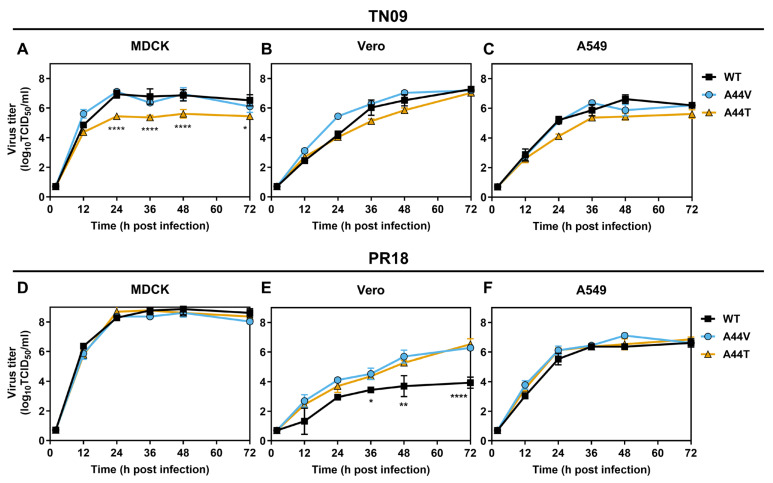
Growth of TN09- and PR18-based viruses in MDCK, Vero, and A549 cells. Cells were infected with viruses at an MOI of 0.01 PFU/cell and incubated at 37 °C. Supernatants were collected every 12 h, and virus titers were measured in MDCK cells by TCID_50_ assay. (**A**–**C**) Growth of TN09 viruses in MDCK (**A**), Vero (**B**) and A549 (**C**) cells. (**D**–**F**) Growth of PR18 viruses in MDCK (**D**), Vero (**E**) and A549 (**F**) cells. Data is combined from three biological replicates. The error bars are geomean with geometric standard deviation. The statistical analysis was done by Two-way ANOVA with Tukey’s multiple comparison test. * *p* < 0.05, ** *p* < 0.01, **** *p* < 0.0001.

Virus replication was measured in MDCK and Vero cells, which are used to produce GMP-grade influenza virus, and in A549 cells, a human lung adenocarcinoma cell line. Viruses were inoculated at an MOI of 0.01 PFU/cell, and supernatants were collected every 12 h for 3 days to measure viral growth by TCID_50_ assay. MDCK cells typically produce high titers of pH1N1 viruses, even those with varying HA stability [31,32,33]. In MDCK cells, TN09-A44V, PR18-A44V, and PR18-A44T replicated similarly to their respective WT viruses, whereas TN09-A44T titers were reduced by approximately 20-fold (Figure 4A,D; Table 3). In Vero cells, destabilizing HA mutations in H1N1 viruses have been reported to support higher replication than WT [34,35,36]. In the present work, all three TN09 viruses replicated similarly in Vero cells (Figure 4B), while PR18-A44V and PR18-A44T reached titers about 300-fold higher than PR18-WT (Figure 4E; Table 3). In A549 cells, replication kinetics of WT and mutant viruses were similar (Figure 4C,F).

### 3.4. Destabilizing HA Mutations Reduce Pathogenicity in Mice

**Figure 5:**
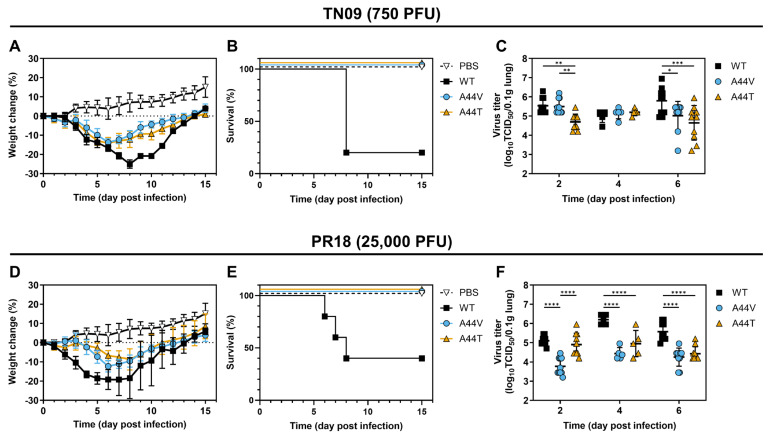
Infection of TN09- and PR18-based viruses in DBA/2J mice. DBA/2J mice were infected with TN09 (750 PFU) or PR18 (25,000 PFU) viruses. Clinical symptoms were monitored for 15 days so that body weight change (**A**,**D**) and survival rate (**B**,**E**) could be calculated. (**C**,**F**) Viral loads in lungs used the WT group as reference for comparison to mutant groups. Tissue was harvested at 2, 4, 6 dpi so that it could be homogenized and titers measured by TCID_50_ assay using MDCK cells. The statistical analysis was done by two-way ANOVA followed by Tukey’s multiple comparison test. * *p* < 0.05, ** *p* < 0.01, *** *p* < 0.001, **** *p* < 0.0001.

Mutations affecting HA stability have been reported to alter pH1N1 influenza virus replication and virulence in mice [31,32,37]. In this study, groups of DBA/2J mice were intranasally inoculated with 750 PFU of TN09-strain virus or 25,000 PFU of PR18-strain virus. Mice infected with WT viruses had average weight losses exceeding 20% and mortality rates above 50% (Figure 5A,D). In contrast, mice infected with viruses bearing destabilizing HA mutations had average weight loss values below 15% and no mortality.

Additional groups of mice were inoculated and euthanized at 2-, 4-, or 6-days post-infection (dpi) for lung homogenization and TCID_50_ assay. TN09-A44V had similar lung titers as TN09-WT at 2 dpi and 4 dpi but significantly lower titers at 6 dpi (*p* = 0.0107; Figure 5C). TN09-A44T titers were lower than WT at 2 dpi (5 × 10^4^ vs. 3.5 × 10^5^ TCID_50_/0.1 g) and 6 dpi (4.5 × 10^4^ vs. 6.3 × 10^5^ TCID_50_/0.1 g). A similar trend was observed for the PR18 viruses. Lung viral loads of PR18-A44V were lower than PR18-WT at 2 dpi (5.9 × 10^3^ vs. 1.3 × 10^5^ TCID_50_/0.1 g), 4 dpi (2.7 × 10^4^ vs. 1.6 × 10^6^ TCID_50_/0.1 g), and 6 dpi (1.8 × 10^4^ vs. 3.8 × 10^5^ TCID_50_/0.1 g). PR18-A44T titers were also lower than WT at 4 dpi (8.9 × 10^4^ vs. 1.6 × 10^6^ TCID_50_/0.1 g) and 6 dpi (2.7 × 10^4^ vs. 3.8 × 10^5^ TCID_50_/0.1 g) (Figure 5F). Overall, destabilizing HA mutations were associated with reduced viral replication in the lungs and decreased pathogenicity in mice.

### 3.5. Impact of HA2-A44V on the Replication and Transmissibility of PR18 Virus in Ferrets

A relatively stable HA protein (activation pH < 5.6) has been shown to be necessary for influenza virus airborne transmission between ferrets [37,38,39,40,41] and from pigs to ferrets [42]. To minimize animal use, we studied the impact of the destabilizing A44V mutation on pH1N1 growth and transmission in only two ferret groups inoculated with either PR18-WT or PR18-A44V. The PR18 strain and the A44V mutation were chosen because a previous study investigated the effect of HA2-A44V on the growth of 2009 pH1N1 in ferrets [24].

**Figure 6:**
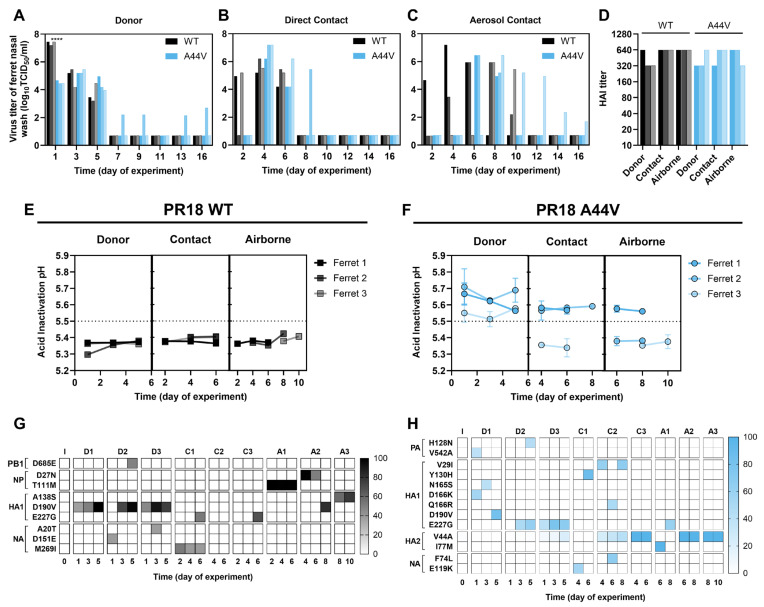
Virus replication and transmission in ferrets. Donor ferrets were inoculated with 1 × 10^6^ PFU of PR18-WT or PR18-A44V viruses. Direct contact ferrets and aerosol contact ferrets were introduced the next day. (**A**–**C**) Virus loads in the nasal cavities of donor ferrets (**A**), direct contact ferrets (**B**), and aerosol contact ferrets (**C**). An unpaired T test with a Gaussian distribution was used for statistical analysis of virus replication in donor ferrets at 1 dpi. **** *p* < 0.0001. (**D**) HAI titers of sera collected at 21 dpi. (**E**,**F**) Virus inactivation pH values of PR18-WT-infected ferrets (**E**) and PR18-A44V-infected ferrets. (**G**,**H**) The heat map shows single-nucleotide variants (SNVs) in collected nasal washes from PR18-WT infected ferrets (**G**) or PR18-A44V infected ferrets (**H**). I: inoculum; D1–D3: donor ferret number 1–3; C1–C3: direct contact ferret number 1–3; A1–A3: Aerosol contact ferret number 1–3.

Donor ferrets (*n* = 3 per group) were intranasally inoculated with 1 × 10^6^ PFU of virus. At 1 dpi, one direct contact ferret was placed in the same cage as one donor ferret, and one airborne contact ferret was housed in an adjacent cage. Nasal wash samples were collected every other day, starting at day 1 of the experiment for donor ferrets and day 2 of the experiment for contact ferrets. At 1 dpi, donor ferrets infected with PR18-WT had nasal virus titers approximately 700-fold higher (*p* < 0.0001) than those infected with PR18-A44V (2.32 × 10^7^ vs. 3.33 × 10^4^ PFU/mL, Figure 6A). Direct contact transmission was detected by day 2 of the experiment in 2 of 3 PR18-WT ferrets and by day 4 of the experiment in the third (Figure 6B). All three direct contact ferrets in the PR18-A44V group had detectable nasal titers at day 4. Airborne transmission occurred at days 2, 4, and 8 in the PR18-WT group and at days 6, 6, and 8 in the PR18-A44V group (Figure 6C). Overall, both viruses achieved 100% contact and airborne transmission (Figure 6D), although transmission of PR18-A44V was delayed in general.

Ferret nasal wash samples were analyzed by virus inactivation assay and whole-genome sequencing (Figure 6E–H). The inactivation pH values of inoculated PR18-WT and PR18-A44V had been previously determined as 5.3 and 5.7, respectively (Table 2). For ferrets inoculated with PR18-WT, nasal wash samples had virus inactivation pH values between 5.3 and 5.45 (Figure 6E), similar to the inoculated virus. In contrast, nasal wash samples from the PR18-A44V group had a broader range of inactivation pH values (Figure 6F). Two of three PR18-A44V airborne-group ferrets (ferrets 2 and 3) had an HA2-V44A reversion mutation (Figure 6H), consistent with their nasal virus inactivation pH values of approximately 5.35 (Figure 6F). The third airborne ferret (ferret 1) had nasal wash pH values of approximately 5.55 and contained HA2-I77M and HA1-E227G substitutions detected at 6 and 8 dpi, respectively (Figure 6H). Overall, two of the three airborne transmission events for PR18-A44V were associated with genetic reversion, and the third was associated with virus stabilization, likely due to the HA2-I77M and/or HA1-E227G mutations.

### 3.6. Effects of HA1-E227G and HA2-I77M on HA and Virus Stability

**Figure 7:**
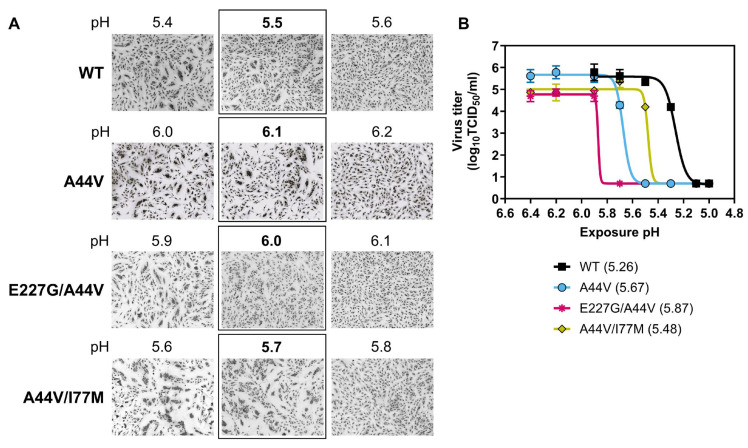
HA and virus stability of PR18-based viruses containing mutations arising after ferret airborne transmission. HA stability was measured by syncytia assay, and virus stability was measured by acid inactivation assay. (**A**) Photomicrographs of syncytia formation in Vero cells infected by PR18-based viruses after treatment with pH-adjusted buffers. HA activation pH was defined as the highest pH causing syncytia and is labelled in the squares on the panels. (**B**) Acid inactivation curves of PR18-based viruses. Virus inactivation pH (labelled in the legend) was calculated by GraphPad Prism 10 as 50% of inhibition using a 4PL curve function. The error bars are geomean with geometric standard deviation.

HA1-E227 is located in the receptor-binding domain adjacent to the receptor-binding site, and HA2-I77 is positioned at the top of α-helix C, forming part of its core at the interface between the head and stem domains (Figure 1A). Based on their structural locations, HA2-I77M was hypothesized to be more likely than HA1-E227G to enhance HA stability. To test this, two new PR18-based viruses were generated, each containing HA2-A44V together with either HA1-E227G or HA2-I77M, and HA and virus stability were measured. Similar to HA2-A44V, the HA1-E227G/HA2-A44V double mutant had a relatively high HA activation pH (6.0) and virus inactivation pH (5.87) (Figure 7). In contrast, the HA2-A44V/HA2-I77M double mutant had lower HA activation and virus inactivation pH values (5.7 and 5.48, respectively), indicating a stabilizing effect.

### 3.7. HA2-I77M Restores Virus Replication and Pathogenicity

**Figure 8:**
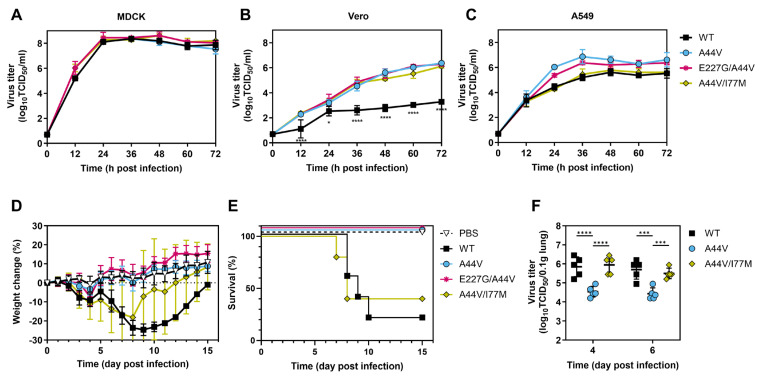
Effects of ferret-adapted mutations on PR18-based virus properties in vitro and in mice. (**A**–**C**) Growth of PR18-based viruses in MDCK (**A**), Vero (**B**) and A549 (**C**) cells. Cells were infected with viruses at an MOI of 0.01 PFU/cell and incubated at 37 °C. Samples were collected every 12h, and virus titers were measured by TCID_50_ assay in MDCK cells. Data was combined from two biological replicates. The error bars are geomean with geometric standard deviation. Two-way ANOVA with Tukey’s multiple comparison test was used for statistical analysis. * *p* < 0.05, **** *p* < 0.0001. (**D**–**F**) The pathogenicity of PR18 viruses in DBA/2J mice. DBA/2J mice were infected with 25,000 PFU of PR18 viruses. The weight change (**D**) and survival rate (**E**) were monitored 15 days after infection. (**F**) The viral load in lungs harvested at 4 and 6 dpi was measured in MDCK cells. The WT group was used as reference for comparison to mutant groups. Two-way ANOVA with Tukey’s multiple comparison test was used for statistical analysis. *** *p* < 0.001, **** *p* < 0.0001.

To determine the effects of the HA1-E227G and HA2-I77M mutations on viral growth in vitro, PR18-based viruses were inoculated into MDCK, Vero, and A549 cells at an MOI of 0.01 PFU/cell, and supernatants were collected every 12 h for 3 days. All viruses replicated similarly in MDCK cells (Figure 8A). The double-mutant viruses HA1-E227G/HA2-A44V and HA2-A44V/I77M had replication kinetics in Vero cells higher than WT but similar to the single-mutant HA2-A44V (Figure 8B).

The PR18-based viruses were intranasally inoculated into DBA/2J mice at a dose of 25,000 PFU. Mice infected with HA2-A44V or HA1-E227G/HA2-A44V lost minimal body weight, whereas those infected with WT or HA2-A44V/I77M lost approximately 20% or more, on average, and experienced 80% and 60% mortality, respectively (Figure 8D–E). In separate groups, mice infected with HA1-E227G/HA2-A44V had lower lung titers at 4 and 6 dpi than WT, while titers from HA2-A44V/I77M-infected mice were similar to WT (Figure 8F). These results showed that the HA2-I77M mutation, which arose in ferrets, likely restored the growth and virulence of HA2-A44V in mice by stabilizing the HA protein and virus.

### 3.8. bNAb Binding by PR18 Virus Containing HA2-A44V/I77M

To evaluate the effect of these mutations on binding of antibodies targeting the HA stem, we generated recombinant wild-type and mutant HA proteins (HA2-A44V, HA1-E227G/HA2-A44V, and HA2-A44V/I77M) and used BLI to evaluate their binding affinities to two bNAbs, CR6261 and P1-05. mAb CR6261 binds to the conserved central stem epitope [18]. mAb P1-05 binds to a conserved epitope on the lower HA stem region called the anchor epitope [30].

A summary of the BLI results is shown in Table 4. Based on the K_D_ values, P1-05 demonstrated high affinity for all four protein constructs, with K_D_ values in the low-picomolar range for all of them. This reveals that the anchor epitope is intact in all mutant HA proteins, suggesting that the mutations do not induce global changes to the HA stem region. In contrast, CR6261 exhibited decreased affinities for mutant HA proteins relative to WT, suggesting that the A44V mutation disrupts the CR6261 epitope. Interestingly, the E227G and I77M mutations, which are outside of the HA stem region, further contributed to a reduction in binding affinity to CR6261, suggesting that these mutations induce more global changes to the HA structure.

**Figure 9:**
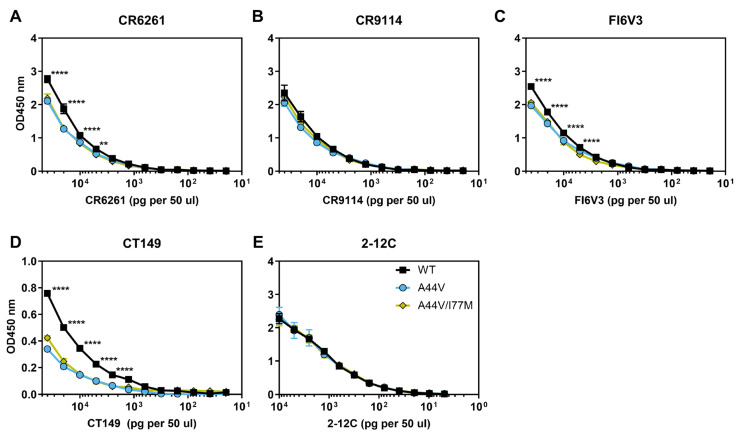
Binding of bNAbs to PR18-based viruses. The binding affinity was analyzed by ELISA assay. CR6261 (**A**), CR9114 (**B**), FI6V3 (**C**), and CT149 (**D**) are broadly reactive antibodies targeting on HA stalk region. 2-12C (**E**) binds to HA globular head as a control. Data was combined from two biological replicates. The error bars are the average with standard deviation. Two-way ANOVA with Tukey’s multiple comparison test was used for statistical analysis. ** *p* < 0.01, **** *p* < 0.0001.

We next used recombinant PR18-based viruses containing these mutations to examine binding to the bNAbs CR6261, CR9114, FI6V3, and CT149 by ELISA, with 2-12C as a control. Consistent with the biolayer interferometry assays using expressed HA protein, ELISAs with recombinant viruses had reduced binding to CR6261 for those containing HA2-A44V and HA2-A44V/I77M (Figure 9A). A similar reduction was observed for FI6V3 (Figure 9C). Binding to CR9114 by the mutant viruses was similar to WT PR18 (Figure 9B), whereas binding to CT149 decreased most for the virus containing the stabilizing HA-adaptive mutation HA2-A44V/I77M (Figure 9D).

In neutralization assays, the IC_50_ values of CR6261 against WT, HA2-A44V, and HA2-A44V/I77M viruses were 73.2, 234.1, and 576.5 ng/50 µL, respectively (Table 5). The IC_50_ values of FI6V3 against these viruses were 71.7, 138.6, and 494.7 ng/50 µL, respectively. Thus, the PR18-HA2-A44V virus containing the ferret-adapted HA2-I77M mutation was more resistant to neutralization by CR6261 and FI6V3 than PR18-WT or HA2-A44V.

### 3.9. Summary of Results

The A44V and A44T mutations destabilized both TN09 and PR18 viruses, increasing HA activation pH from 5.4–5.5 (WT) to 5.8–6.1 (mutant) and virus inactivation pH from 5.3–5.4 (WT) to 5.6–5.7 (mutant). This destabilization reduced H1N1 virus replication and pathogenicity in mice, decreased peak and day-1 nasal wash titers in ferrets, and required an HA-stabilizing mutation to restore airborne transmissibility in ferrets. In the PR18 strain, combining the destabilizing HA2-A44V mutation, which reduces bNAb binding to the central-stem epitope, with a distal re-stabilizing HA2-I77M mutation restored in vivo virus growth and pathogenicity in mice and was concomitant with airborne transmission in a ferret.

## 4. Discussion

Two previous studies showed that an A44V or A44T mutation in the HA stem causes 2009 pH1N1 influenza viruses to become resistant to stem-binding bNAbs [24,26], but it was unknown if the mutations alter HA stability and if bNAb resistance would be maintained in more recently isolated viruses. The 2009 (TN09) and 2018 (PR18) HA proteins studied here differ by 27 amino acids, including HA2-E47K. Early in the 2009 pandemic, the E47K substitution was positively selected in humans [43]. HA2-E47K stabilizes the HA stem by replacing an electrostatic repulsion with HA1-E31 on an adjacent HA protomer to an electrostatic attraction [35] (Figure 1C). Interestingly, HA2 residue 47 is positioned one turn of α-helix A away from the A44V/T bNAb-resistance mutations that were previously shown to alter the conformation of the central stem epitope [24,26]. Here, we found A44V/T increases the HA activation pH and virus inactivation pH values of TN09 by 0.3–0.4 and 0.2 units, respectively. In the context of PR18, which contains the stabilizing E47K substitution, the A44V/T mutations were found to increase HA activation pH and virus inactivation pH by 0.7 and 0.3–0.4 units, respectively. Thus, in the context of the more-stable 2018 virus, which contained E47K, the bNAb-resistance mutations had an even larger destabilizing effect than in the context of 2009 pH1N1. This shows that the individual effects of stability-altering HA mutations may not be additive when the combined mutations are proximal. This suggests that known HA stability-altering mutations may have greater or lesser effects as influenza viruses undergo sequence drift, and this should be monitored.

When comparing WT viruses, PR18 was more than fourfold more sensitive (had IC_50_ values more than four times lower) to neutralization by the bNAbs tested than TN09. Comparing PR18 A44X mutants to PR18 WT, the mutant viruses were approximately two- to threefold less susceptible to bNAb inhibition (had neutralization IC_50_ values more than twice as high), showing the resistance mutations do reduce bNAb potency in a more recent isolate. However, the neutralization IC_50_ values for PR18 viruses containing an A44X mutation were still lower than those of TN09 WT unless containing the additional HA2-I77M mutation, boding well for continued development of stem-directed bNAbs.

Unlike what occurred for the 2012–2013 A/CA/04/2009 (H1N1) human challenge study [21], GMP production of human-challenge influenza viruses should use MDCK instead of Vero cells, and the fidelity of produced virus should be sequence confirmed. Influenza virus titers from MDCK cells typically equal or exceed those from Vero cells [44,45,46]. H1N1 influenza viruses with relatively low HA activation pH values tend to grow to low titers in Vero cells and may acquire HA-destabilizing mutations, as has been shown for A/CA/04/2009 (H1N1) and A/PR/8/34 (H1N1) [22,34]. In the present study, PR18-strain viruses containing destabilizing A44X mutations (HA activation pH 6.1) grew in Vero cells to titers approximately 100-fold higher than PR18 WT (pH 5.4). In contrast, destabilized TN09-strain viruses (HA activation pH 5.8–5.9) grew similarly to TN09 WT (pH 5.5). Thus, introducing destabilizing HA mutations into influenza viruses to increase yields in Vero cells is not expected to be universally effective, let alone desirable, in vaccine manufacture. Although, the introduction of a destabilizing HA mutation has been used to increase yields of A/Kawasaki/173/01 (H1N1), A/Kawasaki/UTK-4/09 (H1N1), A/Yokohama/2013/2003 (H3N2), and human H1N1 live attenuated influenza vaccine viruses in Vero cells [34,35]. In contrast to Vero cells, MDCK cells yield high titers of H1N1 influenza viruses while maintaining vaccine genetic stability, even for H1N1 viruses with high (6.0) or low (5.2) HA activation pH values [31,32,34].

Here, the TN09- and PR18-strain viruses that contained a destabilizing A44X mutation (pH 5.8–6.1) were attenuated in mice compared to their respective WT virus (pH 5.4–5.5). In the backgrounds of both TN09 and PR18 virus strains, the bNAb resistance mutations attenuated virus growth in the lungs of mice, reducing weight loss and eliminating mortality. Also, introduction of the stabilizing I77M mutation into the PR18 virus containing an A44V mutation partially restored HA stability (pH 5.7) and restored virus growth and virulence in mice. This is consistent with previous work. Seasonal and pandemic H1N1 viruses with HA activation pH of approximately 5.5 have been shown to have higher lung titers and cause greater pathogenicity in mice than those having higher or lower HA activation pH [47,48]. It is currently unknown why H1N1 viruses with destabilized HA proteins (pH of approximately 6.0) are attenuated in mice. Extracellular lung pH in mice ranges from 7.0 to 7.4 before and during infection with the TN09 virus [31]. A TN09 virus containing a destabilizing HA1-Y17H mutation (pH 6.0), which was found to be attenuated in mice, was shown to be inactivated more rapidly than TN09 WT at pH 6.4. However, its environmental stability was found to be comparable to that of WT at pH 7.0 [31]. Thus, a pH1N1 virus with an HA activation pH of approximately 6.0 or less is unlikely to be attenuated in the lungs of mice due to extracellular inactivation.

In ferrets, the HA-destabilizing A44V mutation reduced PR18 day-1 nasal wash titers and peak titers. A genotypic reversion (V44A) or a secondary stabilizing mutation (I77M) was required for airborne transmission and either of these changes increased HA and virus stability (decreasing HA activation pH and virus inactivation pH). A relatively stable HA protein (pH < 5.6) has been associated with the adaptation of H1N1 viruses to humans [29,37,49], and increasing experimental evidence shows that HA-stabilizing amino-acid substitutions enhance airborne transmission in ferrets. For example, an H10N7 virus isolated from a seal had higher airborne transmissibility in ferrets than a related mallard isolate, and the increased transmissibility was linked to HA-stabilizing residues [50]. For mixtures of stabilized and destabilized gamma-clade swine A/H1N1 viruses inoculated into ferrets, only viruses with a stabilized HA protein were airborne transmitted [40,51]. For TN09 pH1N1, transmission of virus from a group containing a destabilized mutant (HA1-Y17H, pH 6.0) occurred later than for the WT (pH 5.5) only after re-stabilizing amino-acid substitutions arose in the transmitted virus [42]. A destabilizing HA1-Y17H mutation was also shown to decrease H9N2 virus growth, transmission, and susceptibility in ferrets [52]. For human H3N2 vaccine reference viruses, a destabilizing L194P mutation from egg adaptation prevented airborne transmission in ferrets unless a compensatory HA-stabilizing reversion mutation emerged [29]. Finally, for H5N1 influenza viruses, HA-stabilizing mutations were found necessary, but not sufficient, to enable airborne transmission in ferrets [38,41,53,54]. Recently emerging bovine-origin H5N1 influenza viruses appear to have relatively unstable HA proteins [55], and this may contribute to their limited airborne transmissibility in ferrets [56]. Collectively, these findings show that HA acid stability plays a critical role in influenza virus transmissibility.

If buried HA stem mutations that cause stem-epitope remodeling also destabilize the HA protein, then bNAb-resistant viruses may lose transmissibility. In the present work, such loss-of-function was overcome in one ferret by a second-site, re-stabilizing mutation HA2-I77M. Thus, our data indicate an evolutionary trade-off between the acquisition of bNAb resistance (conferred by HA2-A44V/T mutations) and the maintenance of transmissibility (requiring compensatory mutations such as HA2-I77M). Residue I77M is located at the top and core of α-helix C at the stem-head interface, distal to the central stem epitope (Figure 1A). I77M is a naturally occurring variation found in pH1N1 clade 6B.1A.7, a minor subgroup that was circulating in 2018–2019 [37,38,39]. Analysis of Global Initiative on Sharing All Influenza Data (GISAID) sequences from 2009 to 2024 identified 22 human isolates carrying HA2-A44V/T mutations, although none carried both A44V/T and I77M simultaneously. Nevertheless, stabilizing mutations have been identified throughout the HA trimer, in both HA1 and HA2, at sites that undergo significant secondary and tertiary structural changes during low-pH-induced refolding [48,57,58,59]. This suggests that additional distal mutations may arise to re-stabilize HA after stem-destabilizing resistance mutations, warranting further investigation. A previous study showed that the additivity of HA stability-altering mutations in the stem domain is context-dependent, with some pairs of mutations having additive effects on overall stability [60]. Therefore, stem-epitope remodeling could be achieved by various combinations of mutations that alter epitope structure alongside compensatory stability mutations.

A requirement for two mutations to confer resistance to bNAbs while preserving virus fitness would reduce the likelihood of their emergence. Moreover, different bNAbs target distinct HA regions, including multiple sites in the stem [61]. Thus, combination bNAb therapy targeting multiple epitopes (such as in the stem, monomer interface, and receptor binding site) could further decrease the chance of resistant viruses arising. Additionally, vaccination with chimeric HA proteins has been shown to elicit polyclonal antibody responses targeting diverse HA stem epitopes, which remain protective in mice even against viruses carrying bNAb escape mutations in the stem [26]. Continued monitoring of bNAb resistance mutations and their impact on virus fitness is essential to ensure bNAb efficacy.

Based on previous work and the results described here, several recommendations can be made for future vaccine design and therapeutic strategies that target the HA protein. First, Vero cells should not be used to GMP-grow influenza live-virus vaccines and human-challenge viruses as such conditions have been shown to select for undesired HA-destabilizing mutations, at least in the context of H1N1 viruses. Instead, MDCK cells should be favored for influenza virus production as these cells consistently produce high virus yields and accommodate a broad range of HA stability. Second, GMP-produced virus for human challenge studies should be subjected to genotypic and phenotypic analysis (including HA and virus stability studies) before use in human clinical trials. Third, the effects of HA resistance mutations on HA and virus stability should be examined using multiple influenza virus strains that vary phylogenetically as such effects have been shown to vary by strain-dependent context. Fourth, if a resistance mutation causes the HA protein of a human-adapted influenza virus to become destabilized above an activation pH of approximately 5.5, the potential impact of such a mutation should be considered less of a concern if the mutation reduces virus transmissibility. Conversely, if a resistance mutation stabilizes the HA protein of an emerging influenza virus, bringing its HA activation from a value from above pH 5.5 to below 5.5, then it will be essential to determine if such a resistance mutation promotes virus airborne transmissibility in animal models such as ferrets and/or guinea pigs.

In conclusion, HA2 mutations at residue A44 that cause resistance to stem-binding bNAbs were found to destabilize the HA protein, resulting in increased susceptibility of influenza viruses to inactivation by treatment with mild acid, virus attenuation in mice, and reduced airborne transmissibility in ferrets. The pairing of an HA destabilizing bNAb resistance mutation (i.e., HA2-A44V0 with a distal, re-stabilizing mutation (i.e., HA2-177M) resulted in a regain-of-function of virulence in mice and airborne transmission in ferrets. Thus, the work reveals a potential pathway to sustainable resistance to HA stem-binding bNAbs. Future work should address the potential impact of mutations at position 44 and other buried residues in α-helix A on the susceptibility to bNAbs that bind the central stem epitope in the contexts of other influenza virus subtypes. Moreover, the abilities of HA-stabilizing mutations other than I77M to restore HA stability while maintaining bNAb resistance should be studied.

## Figures and Tables

**Table 1:** Neutralization IC_50_ values of HA stem-binding bNAbs.

Virus	HA (Mutation)	IC_50_ (ng/50 µL)		
		CR6261	CR9114	FI6V3
TN09 ^1^	WT	550.7	446.7	N.D.
TN09	HA2-A44V	760.0	961.6	N.D.
TN09	HA2-A44T	837.8	2023 *	N.D.
PR18 ^2^	WT	73.15	115.7	71.73
PR18	HA2-A44V	234.1 ****	247.2 ***	138.6 **
PR18	HA2-A44T	246.8 ****	250.1 ***	250.1 ****

^1^ A/Tennessee/1-560/2009 (pH1N1). ^2^ A/Puerto Rico/15/2018 (pH1N1). N.D.: not determined. Results are combined data from two biological repeats. IC_50_ values were compared to WT viruses using two-way ANOVA followed by Tukey’s multiple comparison test. * *p* < 0.05; ** *p* < 0.01; *** *p* < 0.001; **** *p* < 0.0001.

**Table 2:** HA and virus stability values for TN09- and PR18-based viruses.

Virus	HA (Mutation)	HA Activation pH	ΔHA Activation pH ^1^	Virus Inactivation pH	ΔVirus Inactivation pH ^1^
TN09 ^2^	WT	5.5	-	5.4	-
TN09	HA2-A44V	5.9	+0.4	5.6	+0.2
TN09	HA2-A44T	5.8	+0.3	5.6	+0.2
PR18 ^3^	WT	5.4	-	5.3	-
PR18	HA2-A44V	6.1	+0.7	5.7	+0.4
PR18	HA2-A44T	6.1	+0.7	5.6	+0.3

^1^ The change of pH value is compared to WT of the same strain. ^2^ A/Tennessee/1-560/2009 (pH1N1). ^3^ A/Puerto Rico/15/2018 (pH1N1).

**Table 3:** Peak titers of viruses in MDCK, Vero, and A549 cells.

Virus	HA (Mutation)	MDCK (TCID_50_/_mL_)	Vero (TCID_50_/_mL_)	A549 (TCID_50_/_mL_)
TN09 ^1^	WT	8.7 × 10^6^	1.9 × 10^7^	4.1 × 10^6^
TN09	HA2-A44V	1.3 × 10^7^	1.6 × 10^7^	2.3 × 10^6^
TN09	HA2-A44T	4.1 × 10^5^	1.1 × 10^7^	4.1 × 10^5^
PR18 ^2^	WT	7.3 × 10^8^	8.6 × 10^3^	4.1 × 10^6^
PR18	HA2-A44V	4.1 × 10^8^	1.9 × 10^6^	1.3 × 10^7^
PR18	HA2-A44T	6.1 × 10^8^	3.4 × 10^6^	7.2 × 10^6^

^1^ A/Tennessee/1-560/2009 (pH1N1). ^2^ A/Puerto Rico/15/2018 (pH1N1).

**Table 4:** Binding affinities of HA proteins for mAbs CR6261 and P1-05.

mAb	HA (Mutation)	Average K_D_ (nM)	X^2^	R^2^
CR6261	WT	0.063	0.051	0.998
	A44V ^1^	0.132	0.021	1.000
	E227G/A44V ^2^	1.60	0.035	0.995
	A44V/I77M ^3^	1.04	0.032	1.000
P1-05	WT	<0.001	0.054	0.999
	A44V	<0.001	0.017	1.000
	E227G/A44V	<0.001	0.021	0.999
	A44V/I77M	<0.001	0.031	1.000

^1^ HA2-A44V. ^2^ HA1-E227G and HA2-A44V. ^3^ HA2-A44V and HA2-I77M.

**Table 5:** HA stability, virus stability, and IC_50_ values of broadly reactive antibodies of PR18-based viruses.

Virus	HA Activation pH	Virus Inactivation pH	IC_50_ (ng/50 µL)	
			CR6261	FI6v3
WT	5.5	5.3	73.2	71.7
A44V ^1^	6.1	5.7	234.1 *	138.6
A44V/I77M ^2^	5.7	5.5	576.5 ****	494.7 ****

^1^ HA2-A44V. ^2^ HA2-A44V and HA2-I77M. The result was from three biological replicates. The IC_50_ was compared with WT viruses using two-way ANOVA followed by Tukey’s multiple comparison test. * *p* < 0.01, **** *p* < 0.0001.

## Data Availability

Data available from the authors upon request.

## Abbreviations

The following abbreviations are used in this manuscript:

4PL curve	four-parameter logistic curve
ANOVA	Analysis of variance
A549 cells	Human lung adenocarcinoma cells
ADCC	Antibody-dependent cellular cytotoxicity
ATCC	American Type Culture Collection
BLI	Biolayer interferometry
bNAbs	Broadly neutralizing antibodies
BSA	Bovine Serum Albumin
cDNA	complementary DNA
CHO cells	Chinese hamster ovary cells
DMEM	Dulbecco’s Modified Eagle’s Medium
Dpi	Day post-infection
ELISA	Enzyme-Linked Immunosorbent Assay
EMPEM	Electron microscopy polyclonal epitope mapping
F-12K	Ham’s F-12K (Kaighn’s Modification) medium
FBS	Fetal bovine serum
GISAID	Global Initiative on Sharing All Influenza Data
GMP	Good Manufacturing Practice
HA	Hemagglutinin
HAUs	Hemagglutination units
HEK 293T cells	Human embryonic kidney cells
IC_50_	Half maximal inhibitory concentration
ID_50_	Median infectious dose
IND	Investigational New Drug application
K_D_ value	Dissociation constant
mAb	Monoclonal antibody
MDCK cells	Madin-Darby Canine Kidney cells
MOI	Multiplicity of infection
Opti-MEM	Optimized Minimal Essential Media
PBS	Phosphate-buffered saline
PBST	PBS with 0.1% Tween-20
Pen-Strep	Penicillin-streptomycin
PFU	Plaque-Forming Unit
PR18	A/Puerto Rico/15/2018
SDS-PAGE	Sodium dodecyl sulfate-polyacrylamide gel electrophoresis
SNVs	Single nucleotide variants
TCID_50_ assay	50% Tissue Culture Infectious Dose assay
TMB substrate	3,3′,5,5′-Tetramethylbenzidine
TPCK-treated trypsin	L-(tosylamido-2-phenyl) ethyl chloromethyl ketone (TPCK)-treated trypsin
TN09	A/Tennessee/1-560/2009
UTR	Untranslated region
Vero cells	African green monkey kidney cells
WHO	World Health Organization
WT	Wild type
